# Development and Measurement of Guidelines-Based Quality Indicators of Caesarean Section Care in the Netherlands: A RAND-Modified Delphi Procedure and Retrospective Medical Chart Review

**DOI:** 10.1371/journal.pone.0145771

**Published:** 2016-01-19

**Authors:** Sonja Melman, Ellen C. N. Schoorel, Karin de Boer, Henriëtte Burggraaf, Jan B. Derks, Det van Dijk, Jeroen van Dillen, Carmen D. Dirksen, Johannes J. Duvekot, Arie Franx, Tom H. M. Hasaart, Anjoke J. M. Huisjes, Diny Kolkman, Sander van Kuijk, Anneke Kwee, Ben W. Mol, Mariëlle G. van Pampus, Alieke de Roon-Immerzeel, Jos J. M. van Roosmalen, Frans J. M. E. Roumen, Ellen Smid-Koopman, Luc Smits, Wilbert A. Spaans, Harry Visser, Wim J. van Wijngaarden, Christine Willekes, Maurice G. A. J. Wouters, Jan G. Nijhuis, Rosella P. M. G. Hermens, Hubertina C. J. Scheepers

**Affiliations:** 1 GROW- School for Oncology and Developmental Biology, Department of Obstetrics and Gynaecology, Maastricht University Medical Center, P.O. Box 5800, 6202 AZ Maastricht, The Netherlands; 2 Department of Obstetrics and Gynaecology, Rijnstate Hospital, P.O. Box 9555, 6800 TA Arnhem, The Netherlands; 3 Midwives Zaltbommel en Maasdriel, Hogeweg 141, 5301 LL Zaltbommel, The Netherlands; 4 Department of Obstetrics and Gynaecology, University Medical Hospital Utrecht, P.O. Box 85090, 3508 AB Utrecht, The Netherlands; 5 Department of Obstetrics, Leiden University Medical Center, P.O. Box 9600, 2300 RC Leiden, The Netherlands; 6 Department of Obstetrics and Gynaecology, Radboud University Nijmegen Medical Center, P.O. Box 9101, 6500 HB Nijmegen, The Netherlands; 7 Department of Clinical Epidemiology and Medical Technology Assessment (KEMTA), Maastricht University Medical Center, P.O. Box 5800, 6202 AZ Maastricht, The Netherlands; 8 Department of Obstetrics and Gynaecology, Erasmus Medical Center, P.O. Box 2060, 3000 CB Rotterdam, The Netherlands; 9 Department of Obstetrics and Gynaecology, Catharina-Hospital, P.O. Box 1350, 5602 ZA Eindhoven, The Netherlands; 10 Department of Obstetrics and Gynaecology, Gelre Hospital, P.O. Box 9014, 7300 DS Apeldoorn, the Netherlands; 11 Royal Dutch Organization of Midwives (KNOV), Mercatorlaan 1200, 3528 BL Utrecht, The Netherlands; 12 Department of Epidemiology, Caphri School for Public Health and Primary Care, Maastricht University Medical Center, Maastricht, The Netherlands; 13 Department of Obstetrics and Gynaecology, Academic Medical Center, University of Amsterdam, P.O. Box 22660, 1100 DD Amsterdam, The Netherlands; 14 Department of Obstetrics and Gynaecology, Onze Lieve Vrouwe Gasthuis, Amsterdam, Oosterpark 9, 1091 AC Amsterdam, The Netherlands; 15 Department of Obstetrics and Gynaecology, Atrium Medical Center Parkstad, P.O. Box 4446, 6401 CX Heerlen, The Netherlands; 16 Department of Obstetrics and Gynaecology, Ruwaard van Putten Hospital, P.O. Box 777, 3200 GA Spijkenisse, The Netherlands; 17 Department of Obstetrics and Gynaecology, Jeroen Bosch Hospital, P.O. Box 90153, 5200 ME ‘s-Hertogenbosch, The Netherlands; 18 Department of Obstetrics and Gynaecology, Tergooi Hospital Blaricum, P.O. Box 10016, 1201 DA Hilversum, The Netherlands; 19 Department of Obstetrics and Gynaecology, Bronovo Hospital, P.O. Box 96900, 2509 JH Den Haag, The Netherlands; 20 Department of Obstetrics and Gynaecology, VU University Medical Center Amsterdam, P.O. Box 7057, 1007 MB Amsterdam, The Netherlands; 21 Scientific Institute for Quality of Healthcare (IQ healthcare), Radboud University Medical Center Nijmegen, P.O. Box 9101, 6500 HB Nijmegen, The Netherlands; Hôpital Robert Debré, FRANCE

## Abstract

**Background:**

There is an ongoing discussion on the rising CS rate worldwide. Suboptimal guideline adherence may be an important contributor to this rise. Before improvement of care can be established, optimal CS care in different settings has to be defined. This study aimed to develop and measure quality indicators to determine guideline adherence and identify target groups for improvement of care with direct effect on caesarean section (CS) rates.

**Method:**

Eighteen obstetricians and midwives participated in an expert panel for systematic CS quality indicator development according to the RAND-modified Delphi method. A multi-center study was performed and medical charts of 1024 women with a CS and a stratified and weighted randomly selected group of 1036 women with a vaginal delivery were analysed. Quality indicator frequency and adherence were scored in 2060 women with a CS or vaginal delivery.

**Results:**

The expert panel developed 16 indicators on planned CS and 11 indicators on unplanned CS. Indicator adherence was calculated, defined as the number of women in a specific obstetrical situation in which care was performed as recommended in both planned and unplanned CS settings. The most frequently occurring obstetrical situations with low indicator adherence were: 1) suspected fetal distress (frequency 17%, adherence 46%), 2) non-progressive labour (frequency 12%, CS performed too early in over 75%), 3) continuous support during labour (frequency 88%, adherence 37%) and 4) previous CS (frequency 12%), with adequate counselling in 15%.

**Conclusions:**

We identified four concrete target groups for improvement of obstetrical care, which can be used as a starting point to reduce CS rates worldwide.

## Introduction

There is a worldwide rise in caesarean section (CS) rates. Although the Netherlands has a relatively low CS rate (16.7%) compared to the United Kingdom (24.6%) and United States (32%), the most impressive rise in CS rate is found in ‘low risk pregnancies’: healthy women with a singleton in cephalic position at term [1–4]. The World Health Organization estimates a CS rate between 10–15% to be optimal [5]. Although a CS is a relatively safe procedure, it is associated with increased short term morbidity and mortality, with an increased risk of abnormal placentation and uterine rupture in future pregnancies [6, 7]. Furthermore, rising CS rates are not associated with improved outcome for mother and neonate [8, 9, 10]. A CS costs twice as much as a vaginal delivery, (1256 euro to 9652 euro extra depending on the country of origin [11]. Adding all costs of future morbidity and increased risk of future repeat CS, the estimated additional costs of one CS are 7500 euro.

The cause of the increasing CS rate is still unknown. Previous studies mostly focus on epidemiological data such as rising maternal age, maternal request for CS and decline in attempt of vaginal birth after CS [12, 13]. Applying the Robson Ten Group Classification system, others mainly identified the nulliparous single cephalic term pregnancy, as well as the women with a previous CS to be important contributors to the total CS rate [14, 15, 16]. This neither reflects appropriate obstetrical care, nor shows which women need improvement of care. Our hypothesis is, that incomplete adherence to guidelines regarding the decision when to perform a CS might be an important explanation for the rising CS rate. This hypothesis is supported by hospital-level variation in CS rates, which cannot be explained by socio-demographic or clinical factors [12].

In recent decades, several international obstetrical organizations have developed evidence-based guidelines with recommendations for optimal care regarding the decision when to perform a CS. However, the crucial issue remains whether these recommendations are actually followed. In order to improve current CS care, it is of importance to gain insight into the extent of guideline implementation in daily practice. Before this can be measured, valid quality indicators for optimal care have to be systematically developed [17, 18].

In the present study, we apply a systematic method for development of evidence-based obstetrical quality indicators. Based on these indicators, we compare current Dutch care to optimal care as described in international evidence-based guidelines. This will allow the identification of target groups of women in which a tailor-made implementation strategy might improve care and reduce CS rates.

## Methods

### Development of CS quality indicators

A systematic RAND-modified Delphi method was used to select a set of key recommendations appropriate for transcription into quality indicators [18]. These recommendations were extracted from national guidelines (NVOG: Dutch Society of Obstetricians and Gynaecologists, CBO: Centraal Begeleidings Orgaan, a Dutch organization aiming at improving the quality of care by health care professionals), international guidelines (RCOG: Royal College of Obstetricians and Gynaecologists, ACOG: American Congress of Obstetricians and Gynaecologists, and SOGC: Society of Obstetricians and Gynaecologists of Canada) and literature [19, 20]. The national expert panel consisted of both obstetricians (N = 13) and midwives (N = 4) and were members of either the Dutch Society of Obstetrics and Gynaecology (NVOG) or the Royal Dutch Organization of Midwives (KNOV). The experts rated and discussed indicators on planned CS (including mode of delivery counselling and CS prevention) as well as indicators on unplanned CS (in an iterative way. The exact procedure for indicator development is described in S1 Text: Description of stepwise procedure of CS quality indicator development.

### Measurement of CS current care

#### Design and setting

We conducted a retrospective multi-center cohort study. This study was situated within the Dutch Obstetric Consortium, which is a research collaboration of obstetric clinics in The Netherlands (http://www.studies-obsgyn.nl).

#### Study population

In order to obtain a representative view of current CS care, this study was conducted in 21 hospitals: 5 university hospitals, 10 non-university teaching hospitals and 6 non-university, non-teaching hospitals located in different regions of The Netherlands. To gain real insight in current obstetrical care and measure quality indicator adherence, women with a CS and women with a vaginal delivery (VD) were included. For example, consider the situation of breech presentation. In such a situation, an external cephalic version should be offered. In order to study guideline adherence, all women with a fetus in breech presentation after 34 weeks need to be identified. In this case, adequate care is offering external cephalic version to women with a breech presentation after 34 weeks, independent of their acceptance or the result of the attempt.

Per hospital, the medical charts of 50 consecutive women who underwent a caesarean delivery as well as a random selection of 50 women who underwent a VD in the same period were analysed. Since it was inefficient to include and analyse all women with a VD, a randomization list was developed per hospital based on the local CS rate. For example, if the local CS rate was 20%, VD sampling rate was 0.25 (0.25*80% = 20%) resulting in the random selection of 50 VD per hospital. We excluded cases with major fetal abnormalities (defined as ‘abnormalities that interfere with standard obstetrical care or vaginal birth’), birth prior to 24 weeks of gestation and fetal demise prior to onset of delivery. Since the Medical Ethical Committee (CMO) of Maastricht (azM/UM) declared that no ethical approval was necessary for this study protocol, no informed consent was required. The patient data were anonymized.

#### Data collection

Trained research nurses from the Dutch Obstetric Consortium gathered the data. We extracted basic obstetrical data for all women from their individual medical charts; including data on previous deliveries (previous VD, CS) and current pregnancy (parity, singleton/ multiple gestation). Furthermore, we gathered indicator specific data for all women to enable calculation of adherence to each indicator. These data included conditions that might influence mode of delivery and existed prior to delivery (diabetes, hypertension) or developed either during pregnancy (suspected fetal macrosomia, intrauterine growth restriction) or during delivery (suspected fetal distress, non-progressive labour). Indicator specific data included ultrasound results, mode of delivery counselling, delivery specifics (e.g. use of ST-analysis or fetal scalp blood sampling, pain medication and oxytocin). We assessed indicator specific data for all women in order to evaluate whether care was provided according to guidelines.

#### Sample size

We assumed a mean adherence to the guidelines of 75%, an alpha of 0.05, and a precision of the estimation of 5%. Next, we assumed an intra-cluster correlation (ICC) of 0.2 and 80 professionals in 20 hospitals. Taking clustering of data across clinicians and within obstetrical departments into account, 960 medical charts were needed for analyses. In order to compensate for loss to follow-up or incomplete data, at least 1,000 women with a CS needed to be included.

In order to enable the calculation of specific events (frequencies) as described in ‘measurement of CS quality indicators’, a random selection of 1000 women with a vaginal delivery were included. This resulted in the analysis of 50 women with a VD and 50 women with a CS per hospital. Based on the local CS rates, the sample size for VD was 24%.

#### Statistical analysis

To assess guideline adherence, performance scores per indicator were calculated, ranging from 0 to 100%. This was done as follows: the number of women to whom the indicator applied and actual care was consistent with the indicator (numerator) was divided by the total number of women to whom the indicator applied (denominator). When an indicator was composed of aggregated items (e.g. the indicator ‘request for CS without medical grounds’), we calculated additional sub percentages for each item. In this case, sub percentages were calculated for 3 additional items: 1. explore reason for CS request, 2. discuss (dis)advantages to CS delivery and 3. offer psychological counselling in case of fear of delivery. Analyses were performed using SPSS statistics 21.

Percentages were weighted for the hospital-specific sampling fractions used in sampling VDs. For example, if a sampling fraction of 0.25 was used in a particular hospital, data for each VD from that hospital counted four (1/0.25) times in the calculation of numerators and denominators.

## Results

### Development of CS quality indicators

**Fig 1:**
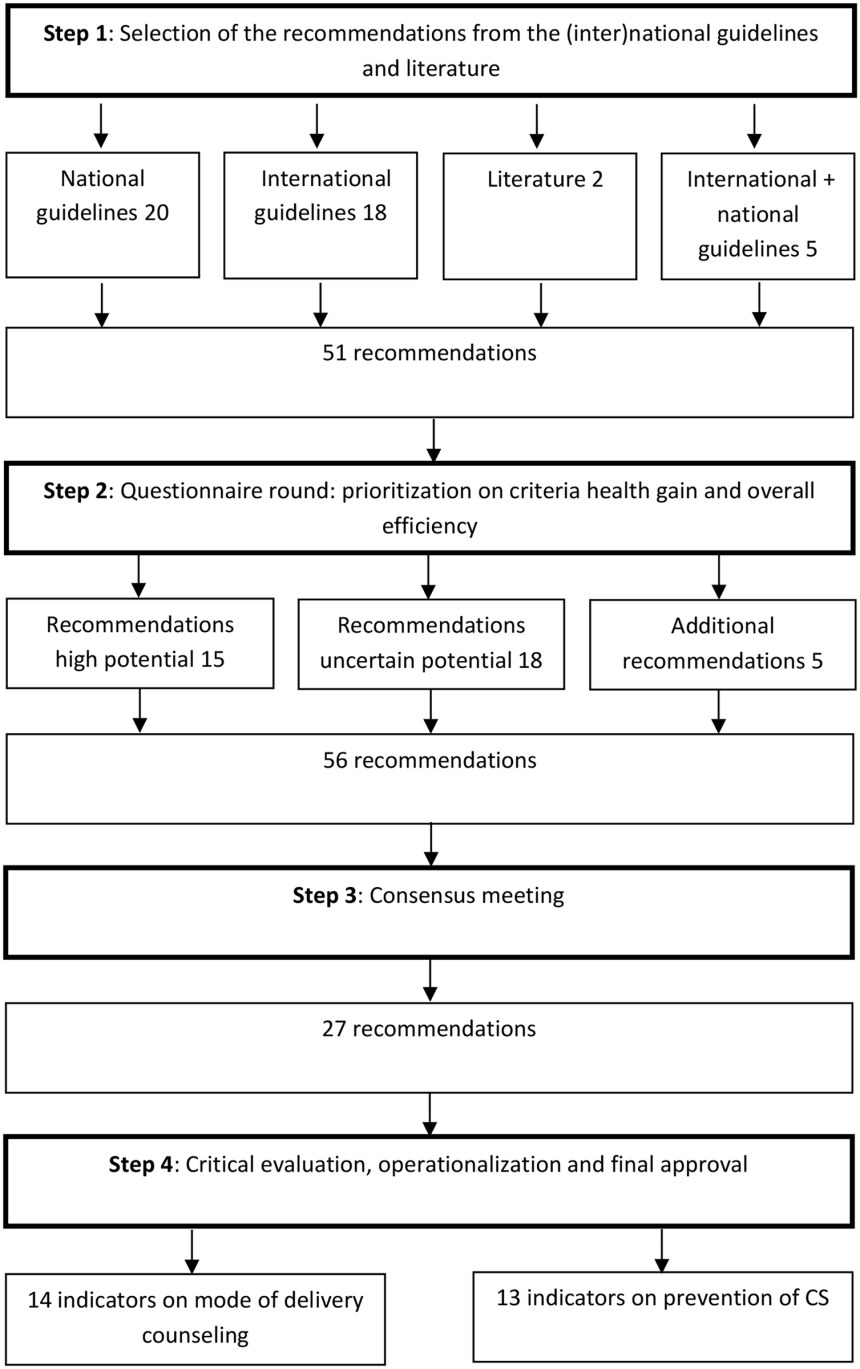
Stepwise procedure of CS quality indicator development.

Based on 51 recommendations, extracted from the guidelines, the stepwise procedure of indicator development resulted in a set of 27 CS quality indicators, including 16 indicators on planned CS (mode of delivery counselling (CS versus VD) and prevention) as well as 11 indicators on unplanned CS. The stepwise procedure of CS quality indicator development is given in Fig 1. The final indicator set is given in Table 1. The indicators on planned CS are shown in S1 Table: Quality indicators on planned CS.

**Table 1:** Set of CS quality indicators.

**1) Quality indicators on planned CS**
***A*) General counseling, CS is not mentioned (VD is the normal conduct)**
1. Twin pregnancy and first child cephalic position
2. Fetal macrosomia (<4.5kg in maternal diabetes, <5kg no maternal diabetes)
3. Preterm labor, cephalic position
4. Small for gestational age without fetal distress
5. Previous shoulder dystocia without impaired perinatal outcome
***B*) Counselling directed at VD (VD and CS are options, VD is preferred)**
6. Position of the placenta at 1-2cm of the internal os
Request for CS without medical grounds:
7. Explore reason for request
8. Discuss (dis)advantages to CS delivery
9. In case of extreme fear: offer psychological counselling
10. Preterm breech delivery (frank, complete breech)
***C*) Counselling mentioning both VD and CS as equal options**
11. Breech presentation at term
Previous CS (Inform on risks and chance for successful VBAC)
12. Inform on low risk of uterine rupture
13. Inform on high chance of successful VBAC
14. Inform on increased risk and lower success rate in case of need for labor induction
***D*) Prevention of planned CS**
15. Offer external cephalic version in case of non-cephalic position
16. Use of internal audit on CS
**1) Quality indicators on unplanned CS**
17. In case of suspected fetal distress use STAN (ST analysis) or micro blood analysis
In case of non-progressive labor first stage:
18. Rupture of membranes,
19. Urinary catheterization,
20. Use of pain medication, preferably epidural analgesia,
21. Adequate contractions or augmentation of labor
In case of non-progressive labor second stage in nulliparous women:
22. Active pushing recommended,
23. Adequate contractions recommended,
24. Consider vacuum extraction if the head is <1/5^th^ palpable per abdomen
25. Continuous support during labor for women with or without prior training
26. Use of partogram
27. Involvement of consultant obstetrician in decision making for CS

### Study population

All 21 hospitals were asked to provide data of 50 VD as well as 50 CS, which would result in a study population of 2100 women. There were 22 women who met the exclusion criteria. Not every hospital analysed the requested 100 women, resulting in an analysis of 2060 women. When adjusting for the random selection of vaginal deliveries, these 2060 women represent a total study population of 4687 women.

### Measurement of current care

Table 2 shows the frequency of specific obstetrical events, as described by the indicators, as well as the performance scores (indicating adequate care) in the total study population (N = 4687 women) concerning: planned CS and unplanned CS.

**Table 2:** Quality of care measured by CS quality indicator.

**1) Quality indicators on planned CS**	**Frequency of occurrence**	**Performance score (adherence)**
**A) General counseling, CS is not mentioned (VD is the normal conduct)**		
Twin pregnancy and first child cephalic position	1.3%	16%
Fetal macrosomia (<4.5kg in maternal diabetes, <5kg no maternal diabetes)	4.3%	33%
Preterm labour, cephalic position	4.7%	45%
Small for gestational age without fetal distress	3.3%	43%
Previous shoulder dystocia without impaired perinatal outcome	1.1%	22%
**B) Counseling directed at VD (VD and CS are options, VD is preferred)**		
Position of the placenta at 1-2cm of the internal os	0.02%	100%
Request for CS without medical grounds:	1%	
Explore reason for request		80%
Discuss (dis)advantages to CS delivery		66%
In case of extreme fear: offer psychological counseling		62%
Preterm breech delivery (frank, complete breech)	1.7%	1.3%
**C) Counseling mentioning both VD and CS as equal options**		
Breech presentation at term	4.1%	56%
Previous CS (Inform on risks and chance for successful VBAC):	11.7%	4%
Previous CS and medical reason for induction of labour (inform on risks and chance for successful VBAC)	2.2%	18%
**D) Prevention of planned CS**		
Offer external cephalic version in case of non-cephalic position	6%	77%
**1) Quality indicators on unplanned CS**		
In case of suspected fetal distress use STAN (ST analysis) or micro blood analysis	16.9%	46%
In case of non-progressive labor first stage:	11.1%	
Rupture of membranes,		95%
Urinary catheterization,		61%
Use of pain medication		78%
Use of pain medication: epidural analgesia,		49%
Adequate contractions recommended		93%
Before performing a CS, an optimal situation (A-E)>2hrs		23%
Before performing a CS, an optimal situation (A-E)>4hrs		15%
In case of non-progressive labor second stage in nulliparous women:	12.7%	
Active pushing recommended,		98%
Adequate contractions recommended,		72%
Consider vacuum extraction if the head is < 1/5^th^ palpable per abdomen		45%
Continuous support during labor for women with or without prior training	88.3%	37%
Use of partogram	6.9%	54%

#### 1 Planned caesarean section

Table 2 shows that for planned CS, the frequency of the occurrence of the specific events ranged from 0.02% to 11.7% and adequate care (performance scores) ranged from 4 to 100%. Although in many obstetric situations caregivers do not follow guidelines, the impact on total caesarean section rate is not likely to change when the frequency of the situation is very low. This is the case for twin pregnancies with the first fetus in cephalic position, preterm breech and previous shoulder dystocia, occurring in less than 2% in the general population. The population with a high incidence and a low performance are women with a previous CS. In an average obstetric population, 11.7% of all women have a previous CS and in only 15% counselling regarding estimated success rates of a VD, next to risks and benefits involved with CS and VD according to the guidelines was documented. In addition, in only 4% of the medical charts of these women, comments informing on risks and benefits were detailed. The highest performance scores for this group of women were found for mode of delivery counselling in case of placenta position at 1-2cm of the internal os (100%), to offer external cephalic version for non-cephalic position (77%) and counselling on CS without medical grounds (62–80%).

#### 2 Unplanned caesarean section

Unlike the indicators for planned caesarean sections, the indicators for unplanned caesarean sections have a much higher frequency of occurrence, ranging from 11 to 88.3%. In these indicators, guideline adherence in general is higher ranging from 23 to 98%.

Continuous support during labor was advised for all women starting vaginal birth. In 37% of these women the support was actually provided. It was advised to apply additional diagnostics such as ST-analysis or fetal scalp blood sampling to all women with suspected fetal distress, if this was technically possible and no contraindications existed to the procedure. In 46% of the women with suspected fetal distress, additional diagnostics were applied before proceeding to a CS. In women with non-progressive labour, the performance scores of the separate quality indicators (artificial rupture of membranes, urinary catheterization, use of pain medication (preferably epidural analgesia and adequate contractions), ranged from 61% to 95%. However, the expert panel advised to proceed to a CS based on non-progression, not earlier than 2–4 hours after all previous measures were fulfilled. Only in a small proportion of women, these criteria were met and in more than 77% CS were performed to soon.

## Discussion

### Main findings

This study resulted in a set of 27 evidence-based quality indicators on both planned as well as unplanned CS. Current care measurement in the Netherlands identified four major target groups for future implementation strategies due to their high prevalence and low adherence rate: improvement of counselling in women with a previous CS, improvement of implementation of continuous support during labour, additional diagnostics before proceeding to a CS in case of suspected fetal distress and allowing a longer waiting period before proceeding to a CS in case of non-progressive labour.

### Strengths and limitations

Our study offers the first set of CS indicators covering entire obstetrical care, thereby enabling measurement of quality of obstetrical care in situations that exist antepartum (e.g. breech presentation), intrapartum (e.g. non-progressive labour) as well as postpartum (e.g. internal audit on CS). This is in contrast to previously developed indicators which only focussed on peripartum care [21]. We included 21 different types of hospitals in several regions in The Netherlands, analysing more than 1000 women per group in order to determine actual care. When comparing the data from our trial to data from the Foundation Perinatal Registration The Netherlands (PRN: the national obstetrical database), we find that the SC rate is comparable (22.2% versus 23.4%). With a similar distribution among planned and unplanned CS: 11.4%, 10.8% versus 10.5%, 12.9%, respectively [22]. Data from secondary and tertiary care is analysed, since the indicators on CS are not applicable to women who deliver in primary care. Thus, we expect our results to be a good representation of actual care in The Netherlands.

Although a standardised method for the development of quality indicators was used, there are several limitations to this study. To date no study compared the different methods used for quality indicator development. However, the RAND-modified Delphi method offers a systematic approach to indicator development and is a frequently used method that has proven to result in valid quality indicators [23].

In addition, one can challenge whether all quality indicators are usable and accepted in different and specific international obstetrical settings. However, the obstetrical situations described (previous CS, non-progressing labor, suspected fetal distress) are comparable world-wide and the basis of the quality indicators consists of recommendations derived from international guidelines and literature [15, 24]. Although there may be a different approach in obstetrical care in some cases (e.g. preterm breech delivery, fetal blood sampling), we expect a similar approach by most obstetrical healthcare professionals to the identified major categories. Therefore, we believe that the most important quality indicators are likely to be adopted by most obstetrical healthcare professionals internationally.

The data collection from medical charts was performed by trained research nurses from the Dutch consortium, which could introduce bias. It was shown by Luck et al. that medical chart review somewhat underestimates the actual care given [25]. Not every detail of a consultation is noted in the medical chart. Secondly, despite the fact that trained research employees extracted the data, there might be interpretation bias. However, when considering the adherence percentages for the main categories (fetal distress, non-progressive labour and previous CS) in our study, we do not expect our results to change substantially.

### Interpretation

In case of a rare situation like triplets, discussing the necessity of CS will only lead to marginal improvement of general care. However, an improvement strategy will have a considerably larger effect in case of a situation with a relatively high frequency and low adherence rate, such as non-progressive labour. The CS quality indicators allowed us to analyse obstetrical care in the Netherlands, thereby identifying groups of women in whom a high frequency of a certain quality indicator is observed in combination with low adherence. The next step in improvement of care will be to determine factors that influence the mode of delivery decision, by either facilitating or hindering quality indicator adherence. Based on these influencing factors, a tailor-made implementation strategy is expected to have a high impact on obstetrical care. It is essential to evaluate the effect of such a strategy on actual obstetrical care in terms of indicator adherence, CS rate, as well as maternal and neonatal outcome.

An analysis of data from the Consortium on Safe Labor by Boyle et al. showed that the most common indications for a primary CS in their US population were failure to progress (35.4%) and non-reassuring fetal rate tracing (27.3%) [24]. Furthermore, using the same data, Zhang et al found that the a repeat CS rate was a major contributor to the total CS rate (30.9%) [15]. The target groups we identified are commonly mentioned indications for a CS globally, implying that our results are comparable to international data. Ample attention is paid to interventions that might increase trial of labour as well as vaginal birth after caesarean section [26, 27]. There is increasing interest in the prevention of the primary CS, which means CS indications for the nulliparous single cephalic at term are an important factor: non-progressive labor and suspected fetal distress [24, 28, 29, 30]. Therefore, intervention in these groups are likely to have a high impact on current care internationally. The impact of an implementation strategy might vary internationally, depending on local CS rate, size of the target group and local barriers to optimal care. Countries with a high rate of elective repeat CS might benefit more from the same implementation strategy compared to countries with a relatively low CS rate. Not all interventions are applicable internationally. In the United States for example, ST-analysis and fetal blood sampling are not practiced. However, these measures are indicators for good care in our study, and an uptake of these interventions might optimize care.

In general, the cost of a CS is about double that of a vaginal delivery [11]. After a first CS, a large majority of women have a CS in the subsequent pregnancy, ranging from 18 to 72% depending on the country women live in [31]. For each repeated CS, the morbidity increases, with higher risks of operative complications, blood transfusions, IC admittance and hysterectomy, adding extra costs [6–10]. Therefore, with on average one subsequent pregnancy, the extra costs are estimated to approach 7500 Euro. In Europe, 5.2 million women delivered in 2012, with on average a 30% CS rate. With a reduction of 1%, in Europe alone 390 million Euro can be saved. The WHO advocates a CS rate between 10–15%, although this has been challenged. This would result in a cost reduction of 3.9 billion euro every year, with no likely harmful effect on maternal or neonatal outcomes.

Beside the use of the presented indicators for local improvement, a subset could be used for international comparison of CS care. Until now, interhospital and international comparison was directed at classification of CS rates, but this does not reflect quality of care [14, 15, 16, 32]. We believe that comparison of CS care could be directed at the subset of indicators that have the highest impact: 1) in women with a previous CS, structured information on risks and benefits on vaginal delivery compared to planned CS should be given and women should be given a choice; 2) women should be offered continuous support during vaginal delivery 3) before performing a CS for suspected fetal distress, fetal blood sampling or ST-analysis should be performed; 4) before performing a CS for non-progressive labour, a 2–4 hour waiting period should be installed after a situation with ruptured membranes, adequate contractions and adequate pain relief is established. Prior to applying this on an international basis, local current care should be established, as well as local facilitating and hindering factors.

## Conclusions

This study provides a framework for future studies for improvement of guideline adherence and reduction of CS rates, thereby possibly improving the outcome for mother and child. Due to the relatively high frequency of occurrence in combination with a low adherence rate, we identified possible target groups where a tailor-made implementation strategy could improve CS care: offer continuous support during labour, women with a previous CS and women undergoing their primary CS for non-progressive labour or suspected fetal distress. The next step will be to identify barriers and facilitators that influence guideline adherence and incorporate them in an implementation strategy to improve care.

## Supporting Information

S1 Table Quality indicators on planned CS. (DOCX)

S1 Text Description of stepwise procedure of CS quality indicator development. (DOCX)
