# Supplementary material for: Development and Measurement of Guidelines-Based Quality Indicators of Caesarean Section Care in the Netherlands: A RAND-Modified Delphi Procedure and Retrospective Medical Chart Review
Source: PLoS One. 2016 Jan 19;11(1):e0145771. doi: 10.1371/journal.pone.0145771 (PMC4718610; doi:10.1371/journal.pone.0145771)
Supplement: S1 Table — (DOCX) [file pone.0145771.s001.docx]

**S1 Table: Quality indicators on planned CS**

| **Planned CS is advised:** |
| --- |
| Suspected placental abruption and viable fetus |
| Vasa praevia |
| Placenta praevia |
| Placenta localization less than 1cm from the internal os |
| Relevant scar in the uterus (e.g. vertical incision during previous CS) |
| Monoamniotic twin pregnancy |
| Breech presentation at term and maternal pelvic abnormality |
| Breech presentation at term and previous non-progressive labor |
| First genital herpes outbreak in the third trimester of pregnancy |
| Impossible vaginal birth (e.g. due to cervical myomas, congenital malformations) |
| Uterine rupture in previous pregnancy |
| Persistent transverse presentation, despite external cephalic version |
