# Supplementary material for: Development and Measurement of Guidelines-Based Quality Indicators of Caesarean Section Care in the Netherlands: A RAND-Modified Delphi Procedure and Retrospective Medical Chart Review
Source: PLoS One. 2016 Jan 19;11(1):e0145771. doi: 10.1371/journal.pone.0145771 (PMC4718610; doi:10.1371/journal.pone.0145771)
Supplement: S1 Text — (DOCX) [file pone.0145771.s002.docx]

**S1 Text: Stepwise procedure of CS quality indicator development**

**Methods**

The method of indicator development was carried out according to four consecutive steps: 1) identification of recommendations, 2) questionnaire round, 3) consensus meeting and 4) critical evaluation and operationalization.

**Step 1: Identification of recommendations**

Five international guidelines were used to extract recommendations: guidelines of the ACOG (n=1), RCOG (n=3) and SOGC (n=1). In addition, all national guidelines of the Dutch Society of Obstetricians and Gynaecologists (NVOG) with recommendations that may affect CS rates were used (e.g. fetal surveillance during labor, breech presentation, twin pregnancy etc.). Furthermore, the guideline on antiretroviral therapy by the CBO (Centraal BegeleidingsOrgaan), a Dutch organization aiming at improving the quality of care by health care professionals, was selected. In addition, the articles of Silverberg et al. and Robson et al. were used for their information on condyloma treatment in pregnancy and medical audit to reduce CS rates, respectively.^[14, 15]^

Subsequently, the recommendations were categorised as follows: 1) planned CS (including recommendations on counselling on vaginal delivery (VD) versus caesarean section (CS) in different situations and prevention of planned CS) 2) unplanned CS. The recommendations considering counselling include situations in which a) general counselling is advised without mentioning the possibility of choosing CS (VD is the normal conduct); b) counselling is advised directed at VD (VD and CS are both options but VD is preferable; c) counselling is advised mentioning both VD and CS as equal options; and d) planned CS is advised.

**Step 2: Questionnaire round**

The identified recommendations were subdivided according to the abovementioned categories and were transformed into a questionnaire that was sent to the expert panel. If available, the questionnaire contained the evidence level per recommendation in order to support the decision-making process. The experts were asked to rate each recommendation on a nine-point Likert scale with respect to their value for both health gain and overall efficacy. In order to be able to distinguish between recommendations with a high score on the Likert scale, a ranking of recommendations per category was used. Furthermore, the experts were requested to add complementary recommendations. This part of the procedure resulted in an allocation of the recommendations for the consensus meeting into recommendations with high, uncertain or low potential as quality indicator.

Recommendations were considered ‘high potential’ if: 1) the median rating of the recommendation was 8 or more; 2) the recommendation was in the

top ranking of the specific category and had at least 20% of the maximum score and 3) 70% or more of the expert panel ratings were in the highest tertile on the Likert scale (7, 8 or 9). There were three possible appraisal combinations that rated the recommendation as ‘uncertain potential’: 1) a median score of 8 or more and a top ranking for less than 20% of the maximum score; 2) a median score less than 8 and a ranking for at least 20% of the maximum score or 3) a rating of 30% or more in the highest (7, 8 or 9) and lowest tertile (1, 2 or 3) on the Likert scale (=disagreement). The recommendation was determined to have ‘low potential’ if none of the abovementioned criteria were applicable.^[16]^

**Step 3: Consensus meeting**

In a face-to-face consensus meeting the results of the questionnaire round were discussed. In order to improve the debate each expert was provided with the overall results from the questionnaire round, as well as their own ratings and additional remarks. Firstly, the experts were asked if they agreed that the recommendations marked ‘high potential’ indeed had to be selected as potential quality indicators to assess and monitor quality of care, and whether or not they could agree to the dismissal of those recommendations considered ‘low potential’ as quality indicators. In addition, the experts were requested if there were any recommendations marked as ‘uncertain potential’ they would strongly advise to consider as an indicator. The members of the expert panel were finally asked which additional recommendations proposed by an expert would be suitable as quality indicator.

**Step 4: Critical evaluation and operationalization**

After the consensus meeting the results were offered to all the members of the expert panel for final approval. Subsequently, the selected recommendations were translated into indicators by defining numerators and denominators, i.e. the number of women in whom a certain test or intervention should have been performed and has been performed, divided by the number of women in whom a certain test or intervention should have been performed. Using this method, an adherence percentage could be calculated in order to assess current care.

**Results**

**Step 1: Identification of recommendations**

In the first step, 51 recommendations on CS were identified: 16 situations where VD was the preferred mode of delivery (category 1a and 1b); 9 situations in which counselling was advised on the possibility of choosing either vaginal or caesarean delivery (category 1c); 2 recommendations on prevention of planned CS (category 1d), and 14 situations where planned CS was advised. Furthermore, there were 8 recommendations on unplanned CS.

**Step 2: Questionnaire round**

The 17 experts each received a questionnaire, of which 16 were returned (2 non-responders, 94% response rate). Out of the 51 recommendations, 14 had ‘high potential’, 18 had ‘uncertain potential’ and 19 had ‘low potential’. The experts suggested 5 additional recommendations.

**Step 3: Consensus meeting**

Eight experts attended the consensus meeting (47%), 5 obstetricians and 3 midwives. This meeting resulted in a consensus-based set of 27 key recommendations labelled according to two categories [See Table 1]. Category 1 comprised 14 recommendations on mode of delivery counselling: 1a) general counselling is advised without mentioning the possibility of choosing CS (VD is the normal conduct) (N=5); 1b) counselling is advised directed at VD (VD and CS are both options but VD is preferable) (N=5), 1c) counselling is advised mentioning both VD and CS as equal options (N=4). There were 2 recommendations on prevention of planned CS (1d). The experts approved of 11 recommendations on the prevention of unplanned CS (category 2).

With regard to the situations were planned CS was advised (category 1d), the 12 indications were not considered to be of high potential for measuring quality of care. For example, in case of placenta praevia, it is not likely that an obstetrician would proceed with VD. Consequently, it is not expected that adherence to such an indicator would be low, and improvement of care would be necessary.

**Step 4: Critical evaluation, operationalization and final approval**

The 27 key recommendations were sent to the expert panel for final approval, which was obtained from 16 experts; there was 1 non-responder.

Finally, the selected recommendations were translated into indicators by defining numerators and denominators: i.e. the number of women in whom a certain intervention or counselling method should have been performed and has been performed, divided by the number of women in whom a certain intervention or counselling method should have been performed. For example: one of the indicators states that in case of breech presentation after 34 weeks, external cephalic version should be offered. Guideline adherence then is measured by dividing the number of women in the study group with a fetus in breech presentation after 34 weeks in whom external cephalic version is offered by the total number of women with a fetus in breech presentation after 34 weeks.

This stepwise procedure resulted in a set of 27 CS quality indicators, including 16 indicators on planned CS as well as 11 indicators on unplanned CS. The process of development of quality indicators on CS by using the RAND-modified Delphi method is depicted in Figure 1, the final indicator set is given in Table 2.
